# Cryo-EM structure of translesion DNA synthesis polymerase ζ with a base pair mismatch

**DOI:** 10.1038/s41467-022-28644-7

**Published:** 2022-02-25

**Authors:** Radhika Malik, Robert E. Johnson, Louise Prakash, Satya Prakash, Iban Ubarretxena-Belandia, Aneel K. Aggarwal

**Affiliations:** 1 Department of Pharmacological Sciences, Icahn School of Medicine at Mount Sinai, New York, New York, NY USA; 2 Department of Biochemistry and Molecular Biology, 301 University Blvd. University of Texas Medical Branch, Galveston, TX USA; 3 Instituto Biofisika (UPV/EHU, CSIC), University of the Basque Country, E-48940 Leioa, Spain; 4 Ikerbasque, Basque Foundation for Science, 48013 Bilbao, Spain

**Keywords:** Cryoelectron microscopy, Translesion synthesis, Translesion synthesis

## Abstract

The B-family multi-subunit DNA polymerase ζ (Polζ) is important for translesion DNA synthesis (TLS) during replication, due to its ability to extend synthesis past nucleotides opposite DNA lesions and mismatched base pairs. We present a cryo-EM structure of *Saccharomyces cerevisiae* Polζ with an A:C mismatch at the primer terminus. The structure shows how the Polζ active site responds to the mismatched duplex DNA distortion, including the loosening of key protein-DNA interactions and a fingers domain in an “open” conformation, while the incoming dCTP is still able to bind for the extension reaction. The structure of the mismatched DNA-Polζ ternary complex reveals insights into mechanisms that either stall or favor continued DNA synthesis in eukaryotes.

## Abstract (web summary)

The structure of mismatched DNA-Polζ ternary complex provides a basis for understanding what makes Polζ adept at extending DNA synthesis past mismatched base pairs.

## Introduction

Assembled from catalytic Rev3 and accessory Rev7, Pol31, and Pol32 subunits, the translesion DNA synthesis (TLS) polymerase ζ (Polζ) plays an important role in the replication of damaged or mismatched DNA in eukaryotic cells^1–4^, and in the prevention of cancer^5,6^. We recently reported the cryo-EM structures of *S.cerevisiae* Polζ holoenzyme without DNA (4.1 Å) and in the act of DNA synthesis (3.1 Å)^7^. The structures decrypt a pentameric ring-like architecture for Polζ, with the catalytic Rev3 and the accessory Pol31, Pol32, and two Rev7 subunits (Rev7_A_ and Rev7_B_) forming a daisy chain of protein-protein interactions^7^. Rev3 makes contacts to the DNA via its palm, fingers, thumb, exonuclease, and N-terminal (NTD) domains. While in apo Polζ the Rev3 fingers domain adopts an “open” conformation, in the matched DNA bound form it lies flush against the nascent base pair in a “closed” conformation and provides a basis for Polζ’s high fidelity during the nucleotide insertion step^7^. The ability of Polζ to tolerate mismatches and lesions at the primer terminus appears to derive in part from the path divergence of the linker between the NTD and the palm, which creates space to better accommodate deviations from Watson-Crick (W-C) base pair geometry^7^.

**Fig. 1:**
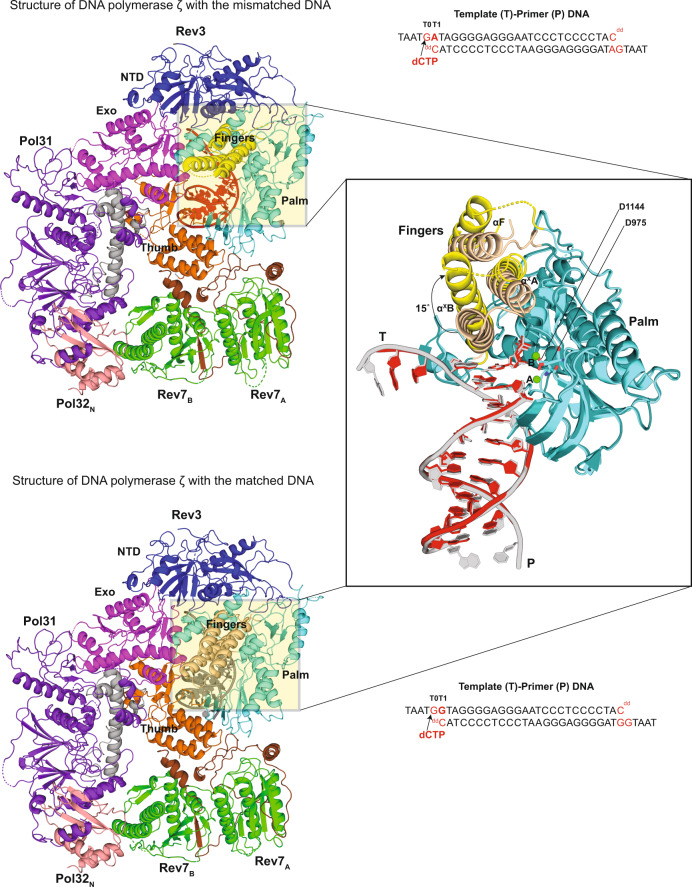
Structure of mismatched (A:C) DNA-Polζ ternary complex. For the mismatched (A:C) DNA-Polζ ternary complex, the NTD (N-terminal domain), Exo (Exonuclease), RIR (Rev7 interacting region), fingers, palm, thumb, CTD (C-terminal domain) of Rev3 are shown in blue, magenta, brown, yellow, cyan, orange, and light gray, respectively; Rev7_A_ and Rev7_B_ in green; Pol31 and Pol32_N_ in purple and salmon, respectively; and the DNA and incoming dCTP in red. For comparison, the matched (G:C) DNA-Polζ ternary complex^7^ (PDB ID: 6V93) is shown below following the same color scheme, except for the fingers domain depicted in wheat color and the DNA and incoming dCTP in gray. The figure also displays the palindromic template-primer DNA sequences and the dCTP used to reconstitute the ternary complexes with Polζ. The right panel displays a close-up view of the region around the DNA, upon an ~90° clockwise rotation of the structure along the y-axis, depicting the fingers helices αF, α^x^A, and α^x^B in a closed (matched DNA complex) and open (mismatched DNA complex) conformation. An arc arrow highlights the ~15^°^outward movement of the fingers domain in the mismatched DNA complex relative to the matched DNA complex. Also highlighted in sticks are the active site residues D975 and D1144. Green spheres depict the active site metal B in the mismatched DNA complex and the active site metals A and B in the matched DNA complex.

We present here, at a nominal resolution of 3.05 Å, a cryo-EM structure of Polζ with an A:C mismatch at the primer terminus (Fig. 1). The structure of the mismatched DNA-Polζ ternary complex provides a basis for understanding what makes Polζ more adept than other eukaryotic B-family DNA polymerases at extending DNA synthesis past mismatched base pairs.

## Results and discussion

### Structure of the mismatched DNA-Polζ ternary complex

The Rev3, Rev7_A_, Rev7_B_, Pol31, and Pol32 subunits are organized around the mismatched duplex DNA in the same pentameric ring-like architecture as the matched complex (Fig. 1), with catalytic Rev3 alone making all of the contacts to the DNA. Rev3 embraces the mismatched template-primer with its palm (residues 329–373; 941–1043; 1098–1215), fingers (residues 302–328; 1044–1097), thumb (residues 1216–1372), inactive exonuclease (residues 662–894) domains, and the NTD (residues 1-301, 374–400, 895–940). The palm, thumb, and the exonuclease domains, as well as the NTD, occupy positions that are essentially identical to those observed with the matched DNA^7^ (Fig. 1). That is, the palm interacts with the replicative end of the template-primer and carries the active site residues (Asp^975^ and Asp^1144^), the thumb grips the duplex portion of the primer-template and makes contacts through the minor groove, the inactive exonuclease domain extends towards the major groove, and the NTD makes numerous contacts with the unpaired portion of the template strand. However, unlike the matched DNA-Polζ ternary complex, the fingers domain of Polζ on the mismatched template adopts an open conformation^8^, wherein the fingers helices αF, α^x^A and α^x^B rotate outwards by ~15° from the palm domain (Fig. 1), reminiscent of their position in the Polζ apo structure^7^ (Supplementary Fig. 1). Interestingly, the fingers helices are less defined in the cryo-EM density compared to the matched complex (Supplementary Fig. 2a), suggesting motion around the open conformation. The replicative end of the mismatched DNA is also less defined in cryo-EM density than the matched complex (Supplementary Fig. 2b), indicating an increase in motion throughout the Polζ active site when containing a mismatch at the primer terminus.

### Polζ active site response to the mismatched DNA

**Fig. 2:**
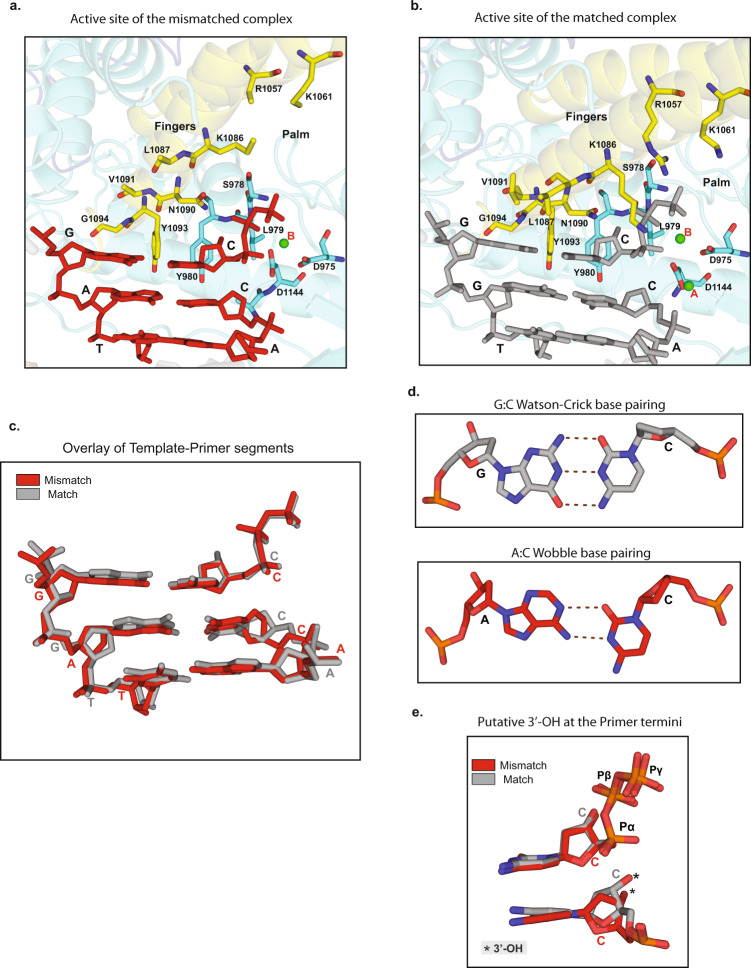
Active sites of the Polζ complexes with mismatched and matched DNA. **a** Close-up view of the Polζ active site in the mismatched DNA complex depicting the key residues from the fingers (yellow) and palm (blue) domains in close proximity to the replicative end of the template-primer DNA (red) and incoming dCTP (red). A green sphere depicts metal ion B. **b** Close-up view of the Polζ active site in the matched DNA complex (PDB ID: 6V93). The coloring scheme is the same as in a., except that the template-primer and dCTP are shown in gray. The complete side chains for few residues including Lys^1061^, Lys^1086^, and Arg^1057^ have not been built in mismatched DNA complex due to less well-defined cryo-EM density. **c** Overlay of equivalent template-primer segments and incoming dCTP for the mismatched (red) and matched (gray) DNA complexes. **d** Base pairing at the primer terminus in the matched (G:C, gray color) and mismatched (A:C, red color) complexes. **e** Position of putative 3’-OH (marked by a star) at the primer terminus relative to the incoming dCTP in the mismatched (red) and matched (gray) complexes.

Intriguingly, even though the fingers domain is open to the same extent as in the apo Polζ structure^7^ (Supplementary Fig. 1), Rev3 is observed with the incoming nucleotide dCTP (position P_0_) opposite the templating base G (position T_0_), establishing standard W-C base pairing for the nascent base pair (Fig. 2a and Supplementary Fig. 3). The dCTP triphosphate moiety treks between the fingers and palm domains, but many of the contacts observed in the matched complex with the fingers helices are lost; including, for example, hydrogen bonds between Lys^1086^ and Arg^1057^ and the α− and γ-phosphates of dCTP, respectively (Fig. 2a, b). The dCTP is anchored loosely in the active site by inter-base hydrogen bonds with templating G, and by the contact it maintains with the palm domain, including stacking interactions between its sugar and Tyr^980^, and hydrogen bonds between its β- and γ-phosphates and the main chain amides of Leu^979^ and Ser^978^, respectively. Of the two metals “A” and “B” observed in the active site of the matched complex^7^ and associated with a two-metal ion mechanism of catalysis^9,10^, only metal B, a Ca^2+^ ion, appears to be coordinated to the dCTP triphosphate moiety (Fig. 2a, b). The absence of metal A likely reflects the overall openness and mobility of the Rev3 active site when accommodating a mismatch at the primer terminus.

An open fingers domain creates space above the nascent G:C base pair and many of the van der Waals contacts observed in the matched complex are lost, including those from Leu^1087^ and Val^1091^ of the fingers helix α^x^B (Fig. 2a, b). But, despite the open conformation of the fingers domain, Tyr^1093^ and Gly^1094^ from helix α^x^B continue to impinge on the minor groove side of the nascent base pair and provide a basis for some fidelity for W-C base pairing at the insertion position (Fig. 2a).

Amongst the various hydrogen bonding schemes that have been considered for an A:C mismatch within a DNA duplex, the DNA cryo-EM density we observe is most consistent with a “wobble” base pairing, in which the N^6^ and N^1^ atoms of adenine make putative hydrogen bonds with N^3^ and O^2^ atoms of cytosine, respectively (Fig. 2c, d). This would be the same configuration described by Kennard and co-workers^11^ for an A:C mismatch in a DNA duplex, implying protonation of N^1^ of adenine. For the wobble pairing, the cytosine base shifts towards the major groove by ~1.2 Å, and the A:C base pair as a whole is much more propeller twisted (−17.8°) than the G:C base pair (−3.8°) in the matched complex (Fig. 2d). The increase in propeller twist (and buckling) extends to the neighboring base pairs, including the nascent base pair. These small perturbations in the conformation of the mismatched DNA duplex appear to hamper the ability of the Rev3 fingers domain to adopt the closed conformation observed in the matched DNA-Polζ ternary complex^7^.

The active site geometry is less primed for the nucleotidyl transfer reaction than in the matched complex. In particular, the A:C wobble pairing results in a shift in the cytidine sugar, which displaces the putative primer 3′OH by ~ 1.2 Å from its position in the matched DNA-Polζ ternary complex^7^ and increases the distance from ~3.8 Å to 4.4 Å to the dCTP α-phosphorous atom - making it less amenable for a nucleophilic attack (Fig. 2e). This increase in distance and the absence of catalytic metal A in the active site may explain the ~20-fold reduction in the ability of Polζ to extend DNA synthesis from mismatched A:C versus matched A:T at the primer terminus^12^.

### What makes Polζ a better extender of DNA synthesis past mismatched base pairs than other eukaryotic B-family polymerases?

It is likely that the overall mobility we observe at the primer terminus (Supplementary Fig. 2), while limiting the rate of catalysis on the one hand, may also facilitate the sampling of catalytically competent conformers for the nucleotidyl transfer reaction. Polζ is also notably different from most B-family polymerases in lacking proofreading exonuclease activity^2–4^, attributed to the absence of catalytic carboxylates in the Rev3 exonuclease domain and by the near absence of a β-hairpin substructure^7^ (Supplementary Fig. 4). The β-hairpin in these B-family polymerase exonuclease domains is postulated to facilitate the transfer of a mismatched primer from the polymerase to exonuclease active site^13,14^, and its near absence in Rev3 may preferably partition the mismatched primer in the polymerase active site for the extension reaction. The mobility of the primer terminus and the near absence of the β-hairpin may combine to lend Polζ the ability to better extend synthesis from mismatched base pairs compared to Polδ or Polε. This is likely coupled to the path divergence of the NTD-palm linker in Polζ, creating extra space for DNA lesions and mismatches^7^. It is striking that despite the DNA distortion from the A:C mismatch and the open fingers domain, the incoming dCTP is still able to bind the Rev3 active site, with its triphosphate moiety oriented in the same manner as in the matched complex (Fig. 2a). Whether the fingers domain remains open or transiently closes during the covalent addition of a nucleotide from a mismatch is an intriguing question that remains to be answered. In either case, the binding/entry of metal A would be a prerequisite for activation of the primer 3’OH for the nucleotidyl transfer reaction. Curiously, an open fingers domain has also been observed in the structure of the human Polα catalytic domain in complex with a DNA duplex and dCTP, and suggested as one reason for the lower fidelity of Polα in incorporating nucleotides after the first dNTP^15^.

It has proven difficult indeed to capture structures of ternary complexes between wild-type B-family DNA polymerases and mismatched duplex DNA. By contrast, the A-family DNA polymerase from *B.stearothermophilus* has been characterized with numerous mismatches, revealing DNA distortions that extend up to six base pairs from the primer terminus^16^. The available structures of replicative B-family polymerase from bacteriophage RB69 with DNA mismatches, for example, have been largely derived with a quadruple mutant of the enzyme designed specifically for low base selectivity^17^. The structure presented here constitutes an early example of how the advent of cryo-EM methods offers unprecedented opportunities to capture and compare B-family polymerases with mismatches for a fuller understanding of the mechanisms that either stall or favor continued DNA synthesis.

## Methods

### Protein expression and purification

*Saccharomyces cerevisiae* Polζ holoenzyme, composed of the full-length Rev3 (residues 1–1,504), Rev7 (residues 1–245), Pol31 (residues 1–487), and Pol32 (residues 1–350) subunits, was expressed in yeast from plasmids pBJ1462 and pBJ1524 and purified as described^7,18^. The complex with the mismatched duplex DNA was prepared by incubating the Polζ holoenzyme in a CaCl_2_ supplemented buffer with an HPLC purified (Integrated DNA technologies) palindromic DNA (in 1.5 molar excess), yielding an A:C mismatch at the extension (T_1_) position (5′-TAATGATAGGGGAGGGAATCCCTCCCCTAC^dd^-3) and G as the templating base. Incoming dCTP was added (1 mM) to capture of Polζ in the act of DNA synthesis past an A:C mismatch.

### Cryo-EM specimen preparation

Specimen preparation for Polζ holoenzyme with the mismatched DNA was done on 300-mesh gold-coated quantifoil grids of 1.2 μm hole size and 1.3 μm spacing. The grids were plasma cleaned using Ar and O_2_ for 8 s using a solarus plasma cleaner (Gatan) prior to loading 2.5 μl of the sample. Back blotting followed by vitrification in liquid ethane was performed with a Leica EM GP2 plunge freezer (Leica microsystems).

### Cryo-EM data collection

The mismatched DNA-Polζ ternary complex was imaged on a Titan Krios microscope (Thermo Fisher Scientific) operated at 300 kV and equipped with a K2 direct electron detector (Gatan) operating in super-resolution mode at a calibrated pixel size of 0.548 Å. The data were subsequently binned by 2 during frame alignment to yield a pixel size of 1.096 Å. Movies were recorded at a frame rate of 200 ms for 10 s for a total accumulated dose of 64.82 e^–^/Å^2^. A nominal defocus range of 0.5–2.5 μm was employed, and the movies were automatically acquired using Leginon to control both the microscope and the K2 camera^19,20^. Frames were aligned using MotionCor2 with dose weighting^21^ and the contrast transfer function (CTF) estimation was performed with CTFFIND4^22^. A total of 2279 images were collected over two sessions and processed to obtain the final three-dimensional (3D) reconstruction (Table 1).

**Table 1:** Summary of structural and refinement statistics.

	Polζ-mismatch (A:C)-dCTP
Data collection and processing	
Magnification	×105,000
Voltage (kV)	300
Pixel size (Å/pixel)	1.096
Electron dose (e^−^/ Å^2^)	64.82
Defocus range (µm)	0.5–2.5
Number of micrographs	2279
Number of particles	120,985
Symmetry imposed	C_1_
Nominal map resolution (Å)	3.05
FSC threshold	0.143
Map sharpening B-factor (Å^2^)	−102
Refinement (phenix)	
Initial model used (PDB code)	6V93
Model resolution (Å)	3.4
FSC threshold	0.5
Model composition	
Non-hydrogen atoms	17,099
Protein residues	2180
DNA/other	24/3
B-factors (Å^2^)	
Protein	79.09
DNA/other	118.32/92.67
R.M.S. deviations	
Bond length (Å)	0.002
Bond angles (°)	0.495
Validation	
Molprobity score	1.74
Clashscore	5.98
Rotamer outliers (%)	0.00
Cβ outliers (%)	0.00
Ramachandran statistics (%)	
Favored	93.87
Allowed	6.08
Outliers	0.05

### Cryo-EM data processing

Particle picking for the mismatch complex was done with FindEM in Appion^23^ using reprojections from the negative-stain reconstruction of Polζ^24^. Particles picked were subjected to multiple rounds of two-dimensional (2D) classification in cryoSPARC2^25^. Selected Polζ particles from about half of the micrographs (Session 1: 1008) were used to obtain a 3D reconstruction with a sphericity of 0.90 out of 1. The other half of the data (Session 2: 1171) was trained with topaz, a neural network-based particle picker^26^ implemented within cryoSPARC2. The micrographs were binned by four and used with resnet8 neural-network architecture. Pi, the expected fraction of positive pixels, was set to 0.027, and the radius parameter, which sets the number of pixels around a labeled particle coordinate, was set to 3. A total of 200 iterations were used to fit the topaz model.

Selected particles from both sessions were combined and iterative rounds of 2D classification were performed. A final set of 210,505 particles was subjected to ab-initio clean-up in cryoSPARC2, which allowed the removal of low-resolution models with preferred orientation. The ab-initio model was refined using non-uniform refinement to generate a 3D reconstruction at a nominal resolution of 3.1 Å based on the Fourier shell correlation (FSC) value of 0.143 between independently refined half sets^27,28^. The cryo-EM map was checked for directional anisotropy (https://3dfsc.salk.edu) and gave a value of 0.967 out of 1. The particles in this reconstruction were subjected to local CTF refinement followed by local refinement within cryoSPARC2, which lead to a 3D reconstruction with an FSC_0.143_ value of 3.05 Å (Table 1 and Supplementary Fig. 5).

### Model building and refinement

Model building was performed in COOT^29^ using the matched DNA-Polζ ternary complex as the initial model (PDB ID: 6V93). The 3D reconstruction was subjected to local map sharpening using the fitted model coordinates in locscale (ccpem)^30,31^ to improve the contrast of the cryo-EM density, especially in the flexible regions of the map. The model was refined by multiple rounds of real-space refinement in Phenix^32^ using the locscale sharpened map. The model was validated using Molprobity^33^ and the data was analyzed in Chimera^34^. The figures were prepared in Pymol^35^.

### Reporting summary

Further information on research design is available in the Nature Research Reporting Summary linked to this article.

## Supplementary information

Supplementary Information

Reporting Summary

## Data Availability

The data that support this study are available from the corresponding author upon reasonable request. The cryo-EM density map generated in this study has been deposited in the Electron Microscopy Data Bank (EMDB) under accession number EMD-24793. The resulting atomic coordinates have been deposited in the Protein Data Bank (PDB) with accession number 7S0T. The atomic coordinates used in the study are available in the PDB with accession numbers 6V93 and 6V8P.
