# Supplementary material for: Cryo-EM structure of translesion DNA synthesis polymerase ζ with a base pair mismatch
Source: Nat Commun. 2022 Feb 25;13:1050. doi: 10.1038/s41467-022-28644-7 (PMC8881453; doi:10.1038/s41467-022-28644-7)
Supplement: Supplementary file 1 — Supplementary Information [file 41467_2022_28644_MOESM1_ESM.pdf]

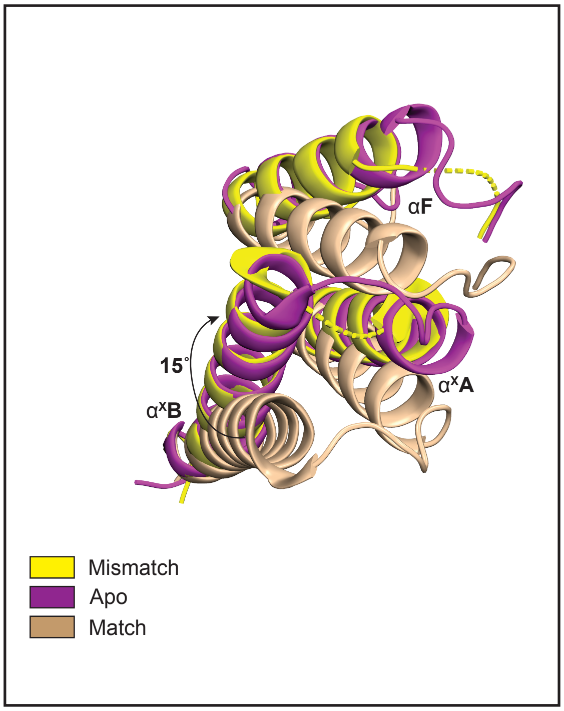


**Supplementary Fig. 1: Overlay of the fingers domain.** The fingers helices of the mismatched DNA-Polζ ternary complex (yellow color) are superimposed with those in apo (magenta color, PDB ID: 6V8P) and the matched DNA-Polζ complex (wheat color; PDB ID: 6V93).

**a. Cryo-EM density for the fingers domain**

Mismatched complex

Matched complex

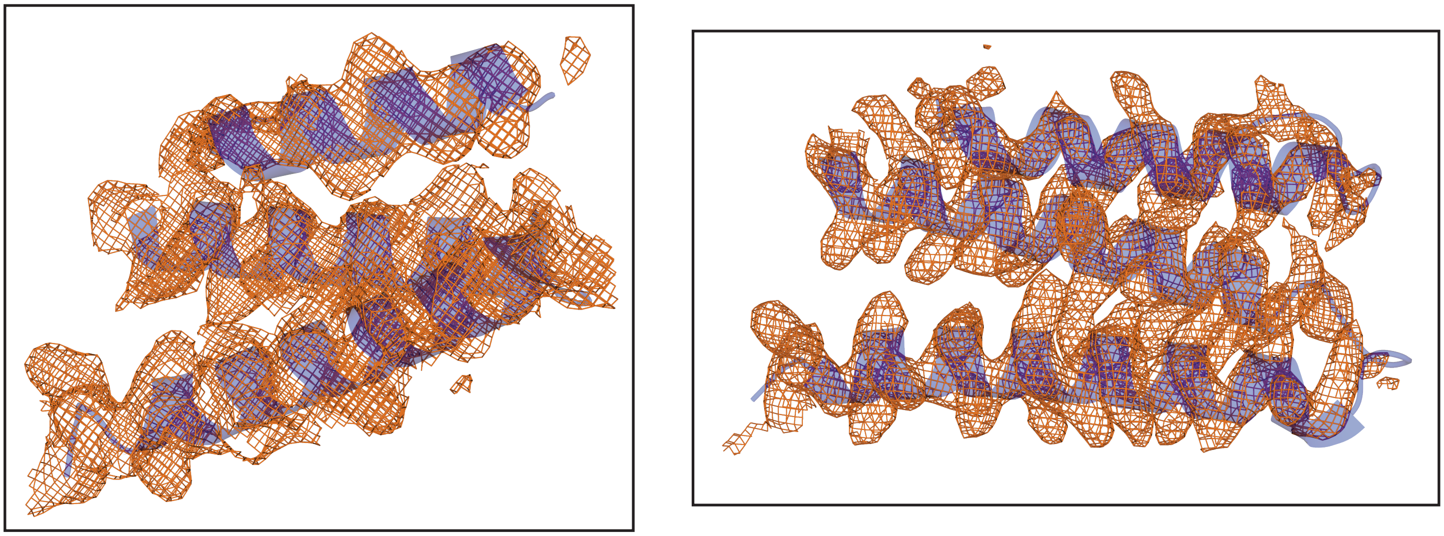

**b. Cryo-EM density for the DNA segments**

Mismatched complex

Matched complex

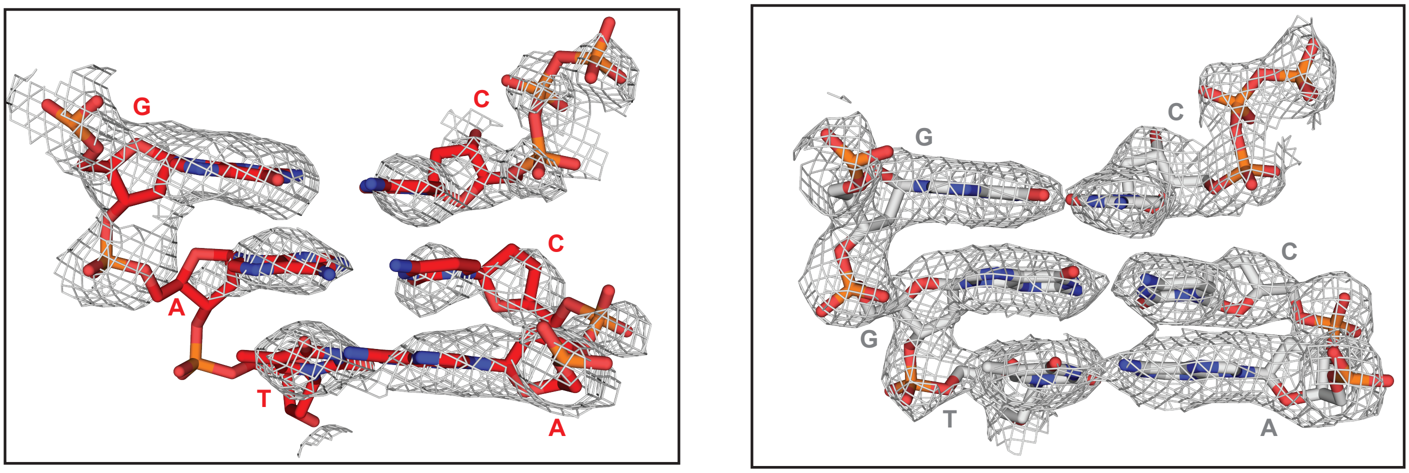


**Supplementary Fig. 2: Comparison of cryo-EM densities from the mismatched and matched DNA-Polζ ternary complexes.** a. Cryo-EM densities for the fingers helices in the mismatched and matched DNA complexes. The helices (blue) are shown in a cartoon format. b. Cryo-EM density for the replicative ends of template/ primer DNA and incoming dCTP for the mismatched (red) and matched (grey) DNA complexes.

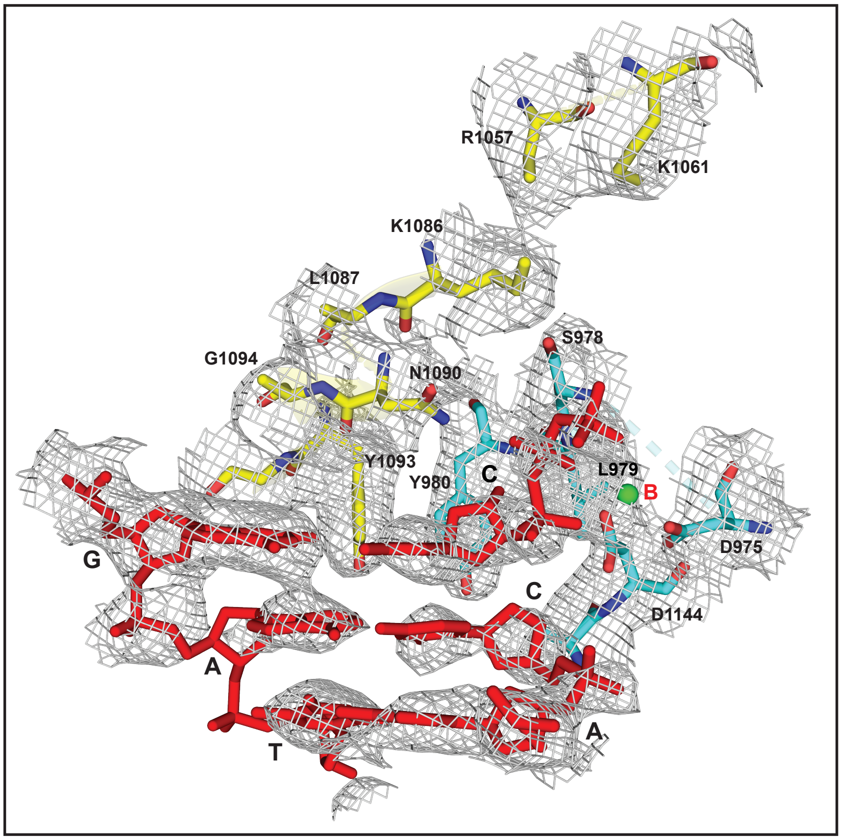


**Supplementary Fig. 3: Cryo-EM density of the active site of the mismatched DNA complex.** Cryo-EM density (grey) of the active site residues, metal, and bases at the replicative end of the template/primer DNA and the incoming dCTP. The active site has been replicated in the same orientation and overall configuration as in Fig. 2a.

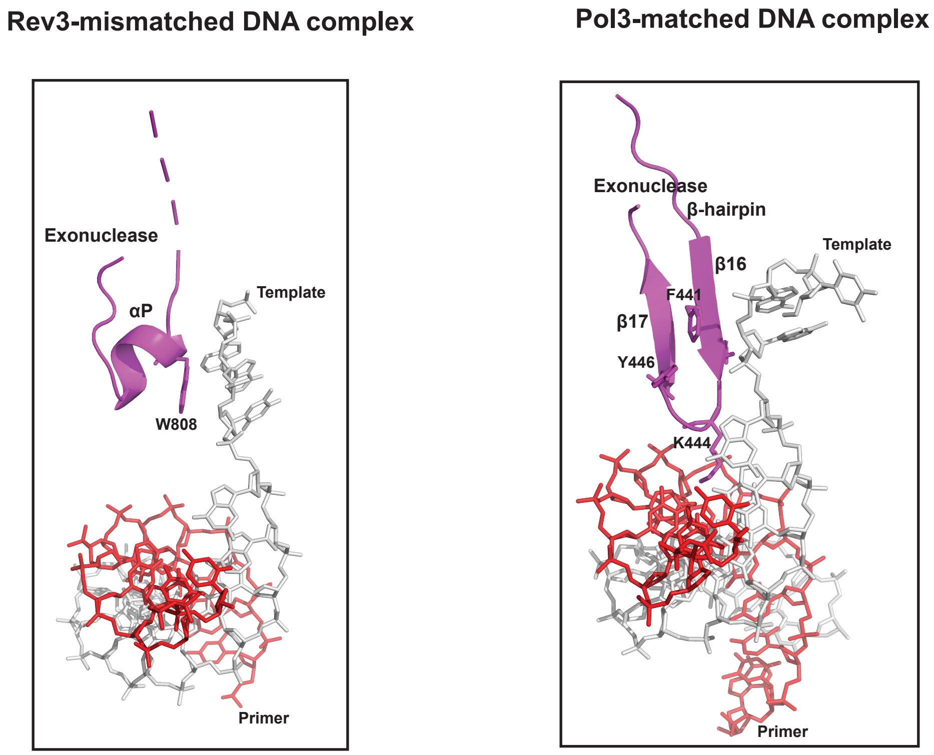


**Supplementary Fig. 4: Substructure/β-hairpin of the exonuclease domain in Rev3 and Pol3**. Comparison of the β-hairpin of the exonuclease domain of DNA-Pol3 complex (PDB ID: 3IAY) (right) with the substructure (a short helical conformation) in the mismatched DNA-Rev3 complex (left). In Pol3, the extended β-hairpin inserts into the DNA major groove and interacts extensively with the unpaired portion template strand. This is consistent with the idea that the β-hairpin facilitates exonuclease activity by helping to hold the template strand in place while the primer strand separates and migrates to the exonuclease active site. In Rev3 the β-hairpin is missing, and is substituted by a short helical substructure that makes far fewer contacts with the template strand.

**a. Representative micrograph**

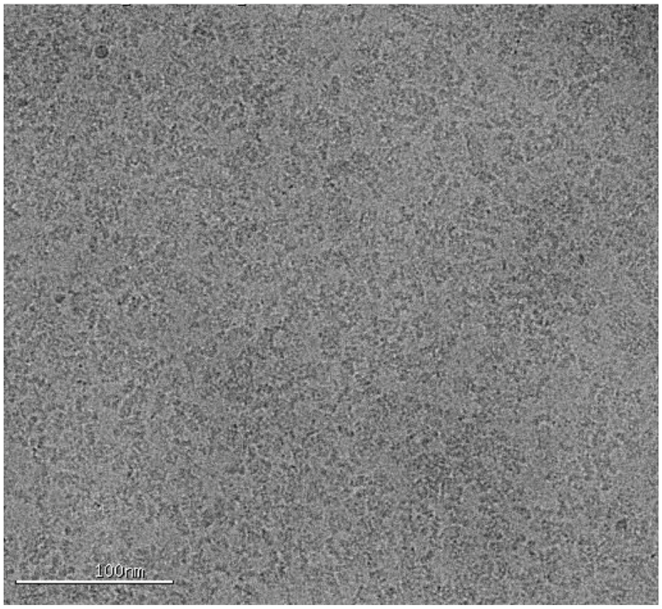


**b. Cryo-EM map of the mismatched complex**

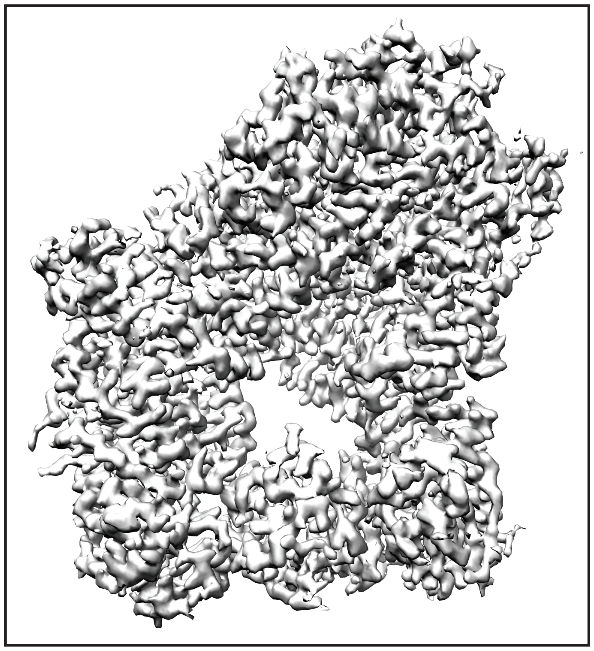

**c. Fourier Shell Correlation (FSC) curves of the cryo-EM map of the mismatched complex**

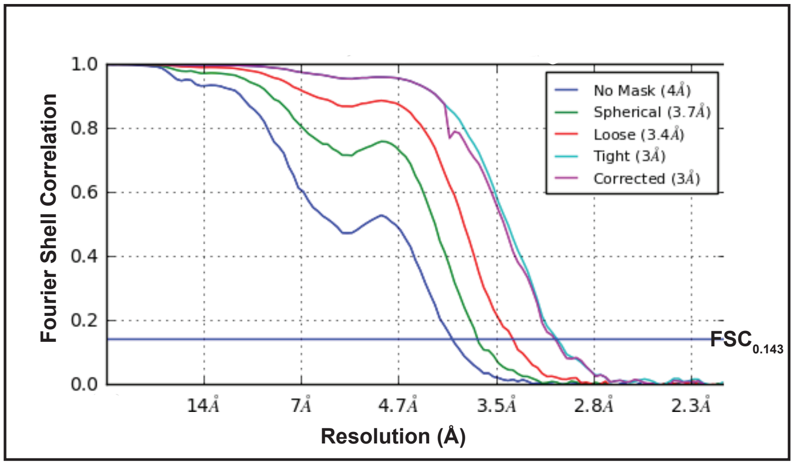


**Supplementary Fig. 5: Cryo-EM map and FSC curves of the mismatched DNA-Polζ ternary complex.** a. Representative micrograph for the mismatched DNA complex is shown b. The cryo-EM density of the full map is shown in grey b. Various Fourier shell correlation (FSC) curves for the mismatched DNA complex derived from variable levels of masking are shown. The $FSC_{0.143}$ value between independently refined sets indicates a nominal resolution of 3.05 Å.
