# Supplementary material for: Cryo-EM structure of translesion DNA synthesis polymerase ζ with a base pair mismatch
Source: Nat Commun. 2022 Feb 25;13:1050. doi: 10.1038/s41467-022-28644-7 (PMC8881453; doi:10.1038/s41467-022-28644-7)
Supplement: Supplementary file 2 — Reporting Summary [file 41467_2022_28644_MOESM2_ESM.pdf]

nature portfolio

Corresponding author(s): Aneel K. Aggarwal

Last updated by author(s): Dec 16, 2021

Reporting Summary

Nature Portfolio wishes to improve the reproducibility of the work that we publish. This form provides structure for consistency and transparency in reporting. For further information on Nature Portfolio policies, see our Editorial Policies and the Editorial Policy Checklist.

Statistics

For all statistical analyses, confirm that the following items are present in the figure legend, table legend, main text, or Methods section.

n/a	Confirmed	
☐	☒	The exact sample size (n) for each experimental group/condition, given as a discrete number and unit of measurement
☐	☒	A statement on whether measurements were taken from distinct samples or whether the same sample was measured repeatedly
☒	☐	The statistical test(s) used AND whether they are one- or two-sided<br>*Only common tests should be described solely by name; describe more complex techniques in the Methods section.*
☒	☐	A description of all covariates tested
☐	☒	A description of any assumptions or corrections, such as tests of normality and adjustment for multiple comparisons
☒	☐	A full description of the statistical parameters including central tendency (e.g. means) or other basic estimates (e.g. regression coefficient) AND variation (e.g. standard deviation) or associated estimates of uncertainty (e.g. confidence intervals)
☒	☐	For null hypothesis testing, the test statistic (e.g. F, t, r) with confidence intervals, effect sizes, degrees of freedom and P value noted<br>*Give P values as exact values whenever suitable.*
☒	☐	For Bayesian analysis, information on the choice of priors and Markov chain Monte Carlo settings
☒	☐	For hierarchical and complex designs, identification of the appropriate level for tests and full reporting of outcomes
☒	☐	Estimates of effect sizes (e.g. Cohen's d, Pearson's r), indicating how they were calculated

Our web collection on statistics for biologists contains articles on many of the points above.

Software and code

Policy information about availability of computer code

Data collection	Leginon v3.3 for data collection
Data analysis	CTFFind4 v3 was used for CTF determination<br>MotionCor2 was used for aligning cryoEM images<br>FindEM in the appion pipeline and Topaz was used for picking particles from EM images<br>cryoSPARC2 were used for 2D classification, 3D classification, EM map calculation<br>Locscale in ccpem was used for local map sharpening<br>COOT v0.9.3 was used for building structure models into the cryoEM map<br>PHENIX v1.16.2-3549 was used for real space refinement of structure against the cryoEM map<br>Chimera v1.15 and PyMOL v2.2.2 were used for analyzing structures and generating figures

For manuscripts utilizing custom algorithms or software that are central to the research but not yet described in published literature, software must be made available to editors and reviewers. We strongly encourage code deposition in a community repository (e.g. GitHub). See the Nature Portfolio guidelines for submitting code & software for further information.

Data

Policy information about availability of data

All manuscripts must include a data availability statement. This statement should provide the following information, where applicable:

- Accession codes, unique identifiers, or web links for publicly available datasets
- A description of any restrictions on data availability
- For clinical datasets or third party data, please ensure that the statement adheres to our policy

The cryo-EM density map generated in this study has been deposited in the Electron Microscopy Data Bank (https://www.ebi.ac.uk/pdbe/emdb/) under accession number EMD-24793. The atomic coordinates have been deposited in the Protein Data Bank (PDB; https://www.rcsb.org) with accession number 7S0T. The atomic coordinates used in the study are available in the PDB under accession codes as accession numbers 6V93 and 6V8P respectively.

Field-specific reporting

Please select the one below that is the best fit for your research. If you are not sure, read the appropriate sections before making your selection.

- [x] Life sciences
- [ ] Behavioural & social sciences
- [ ] Ecological, evolutionary & environmental sciences

For a reference copy of the document with all sections, see nature.com/documents/nr-reporting-summary-flat.pdf

Life sciences study design

All studies must disclose on these points even when the disclosure is negative.

Sample size	No sample size calculation was required. High resolution 2D classes were used as a guide to select the sample sizes for the map calculation.
Data exclusions	No data were excluded from analysis
Replication	Structure of the mismatched DNA bound complex obtained from the cryoEM map compares well with the previously published cryo-EM structures from our group. Cryo-EM density of the mismatched complex compares very well with the matched complex. All the attempts of replication were successful
Randomization	This is not relevant to our study. Reason -All the particles in the dataset are randomly distributed in ice.
Blinding	This is not relevant to our study. Reason- All the steps in the data analysis are independent of each other and do not introduce any kind of bias

Reporting for specific materials, systems and methods

We require information from authors about some types of materials, experimental systems and methods used in many studies. Here, indicate whether each material, system or method listed is relevant to your study. If you are not sure if a list item applies to your research, read the appropriate section before selecting a response.

Materials & experimental systems

n/a	Involved in the study	
x		Antibodies
x		Eukaryotic cell lines
x		Palaeontology and archaeology
x		Animals and other organisms
x		Human research participants
x		Clinical data
x		Dual use research of concern

Methods

n/a	Involved in the study	
x		ChIP-seq
x		Flow cytometry
x		MRI-based neuroimaging
